# Systematic review and meta-analysis of the association between dimensions of inequality and a selection of indicators of Reproductive, Maternal, Newborn and Child Health (RMNCH)

**DOI:** 10.7189/jogh.09.010429

**Published:** 2019-06

**Authors:** Adeyinka E Adegbosin, Hong Zhou, Sen Wang, Bela Stantic, Jing Sun

**Affiliations:** 1 School of Medicine, Griffith University, Gold Coast, Queensland, Australia; 2 School of Information and Communication Technology, Griffith University, Queensland, Australia; 3 Peking University, Beijing, China

## Abstract

**Background:**

Globally, progress in Maternal and Child Health (MCH) has been inconsistent, with several evidence showing both between and within country disparities in several RMNCH outcome measures. In this study, we aim to meta-analyse existing literature on association between three major equity stratifiers and a selection of RMNCH indicators.

**Methods:**

We searched PubMed, Embase, Scopus databases and grey literatures from the WHO, UNICEF and World Bank publications. Using the PRISMA guidelines, we identified and reviewed studies from low and middle-income countries, that explored the effects of inequalities on RMNCH, with focus on studies that utilised data from a nationally representative survey. The review protocol was registered at the PROSPERO international prospective register of systematic reviews.

**Results:**

A total of 28 studies were included in the meta-analysis. Results revealed the existence of marked inequality based on income levels, education and place of residence. The most significant level of disparity was with regards to unmet need for contraception and antenatal coverage. For both respective indicators, those with secondary or higher education were 6 times more likely to have better coverage, than those with lesser level of education; (odds ratio (OR) = 6.25 (95% confidence interval (CI) = 1.68-23.23; I^2^ = 98%, *P* = 0.006) and (OR = 6.17 (95% CI = 3.03-12.56; I^2^ = 97%, *P* < 0.00001) respectively. In contrast, the lowest inequality was in the completion of 3 doses of diphtheria, pertussis and tetanus vaccines (DPT3), those with primary or no education, were equally as likely as those with secondary or higher education to have received DPT3; (OR = 1.21, 95% CI = 0.34-4.27; I^2^ = 96%, *P* = 0.77).

**Conclusions:**

In developing countries, maternal and child health coverage remains highly inequitable and assess to maternal and child health services are governed by factors such as income, level of education, and place of residence.

Progress in both intervention and impact indicators of maternal and child health have been inconsistent over several decades, with several evidences showing both between and within country disparities in several of these RMNCH indicators [1,2]. These disparities are especially significant in Low and Middle-Income Countries (LMICs), with 99% of global maternal and neonatal death occurring in these countries [3].

Furthermore, disparities in access to RMNCH interventions that require fixed health facilities such as antenatal care, skilled birth and postnatal care services tends to be the most significant, when compared to interventions that can be delivered at community or household levels, such as immunisation [4]. Evidence also shows that poor health outcomes at national levels are highly reflective of disparities at individual household levels; such reflection was demonstrated in the work of Chao and colleagues [5], exploring relationships between household wealth quintiles and Under-Five Mortality Rates (U5MR). Access to important health care services, as well as the overall health outcome of women and children, in LMICs often depends on factors such as gender of a child, place of residence, level of education, income and other socio-economic parameters [1]. These factors collectively referred to as ‘dimensions of inequality’, usually have an interwoven effect [2]. Low income level for example, may directly prevent a woman from utilizing health services due to associated medical, non-medical and opportunity costs [3], equally a low level of educational attainment may also imply that there is limited economic opportunities, hence impaired ability to earn sufficient income. Low socio-economic status may also result in women and children living in areas with limited health infrastructure [6].

To effectively assess coverage, and progress in RMNCH, changes in inequality must be monitored over time [7], several indexes and frameworks have been developed for this purpose [7]. These frameworks often comprise of important maternal and child health services and outcome indicators. Examples of these include the composite coverage index [8,9], co-coverage index [10], count down to 2015 indicator framework [11], and the frameworks for monitoring the 2010-2015 [12], and 2015-2030 global strategies [13].

In this study, we explored the eleven core RMNCH indicators developed for monitoring the 2010-2015 global strategy for women’s and children’s health [12]. This framework was appealing to our study, due to its concise, yet widely varied number of indicators and the breadth of existing literature that have explored the various indicators within the framework [13-46].

Despite the substantial body of literature that have explored equity dimensions for the various RMNCH indicators [5,14-46], there remains a significant gap in literature, which concerns the paucity of systematic reviews and meta-analysis synthesizing evidence from the various existing cross-sectional studies. Furthermore, the lack of household survey data for certain interventions implies there are no equity studies for those interventions. For example, no study has investigated inequality in the availability of ARV prophylaxis for pregnant women with HIV/AIDS, this may be explained by the fact that information is not available in any database that allows equity breakdown for this indicator [2]. Likewise, there is a paucity of study on the effect of inequality on Maternal Mortality Rates (MMR), existing studies only examined inequality in maternal mortality at national levels, and not from an individual or household level, for example there are studies examining variation in maternal mortality based on national Gross National Income (GNI) or Gross Domestic product (GDP) levels [15,17], or based on Human Development Index (HDI) [16], no study has examined maternal mortality based on individual household wealth index. This lack of household-level data disaggregation, is also likely due to the fact that maternal mortality is currently estimated through modelling, at national levels only, and equity breakdowns are not available [2].

The objective of our study is to fill the gap concerning the lack of meta-analysis, and to provide a pooled evidence base on the association between a selection of RMNCH indicators, and three important equity stratifiers, with focus on studies that utilised nationally representative data. Other gaps identified regarding the paucity of studies on two of our selected indicators (Maternal Mortality Rates and ARV prophylaxis for pregnant women with HIV/AIDS), could not be filled as it pertains to a lack of survey data for those indicators.

## METHODS

We utilised a framework of eleven core RMNCH indicators from the 2010-2015 global strategy for women’s and children’s health [12], comprising of two mortality indicators, eight coverage indicators and one child nutrition indicators as follows: comprising of eight measures of intervention coverage, and three measures of impact as follows: U5MR, stunting in children under five, Maternal Mortality Rate, unmet need for contraception, Antenatal Care (ANC) coverage, Skilled Birth Attendant (SBA), ARV prophylaxis for HIV positive pregnant women, Postnatal Care (PNC) for mothers and babies within two days of birth, exclusive breastfeeding, three doses of combined diphtheria-tetanus-pertussis immunization coverage (DPT3), and care seeking for suspected pneumonia [13]. Two indicators – Maternal Mortality Rate and ARV for HIV-positive pregnant women were excluded from the analysis, due to lack of household level data.

We categorized the included indicators into two broad groups; negative indicators, and positive indicators. The negative indicators were prevalent among the poor, these includes: U5MR, stunting in children under five, and unmet need for contraception, while the positive indicators are mostly pro-rich, which includes: Antenatal Care (ANC) coverage, Skilled Birth Attendant (SBA), Postnatal Care (PNC) for mothers and babies within two days of birth, exclusive breastfeeding, three doses of combined diphtheria-tetanus-pertussis immunization coverage (DPT3), and care seeking for suspected pneumonia [13]. The review protocol was registered at the PROSPERO international prospective register of systematic reviews. The registration number of the protocol is CRD42018092304 [18].

### Ethics

This study analysed secondary data and was exempted from ethics review by the Griffith University office for research.

### Search strategy

The online databases PubMed, Embase, Scopus were searched until form 2008 until June 2018 for relevant studies, we also search the publications of the World Bank, WHO, and UNICEF. The following key words were used for the search: Gaps OR Disparity AND Maternal Mortality AND Developing Countries, Inequity OR Inequality AND Maternal Mortality AND Developing Countries, Gaps OR Disparity AND Under-five Mortality AND Developing countries, Inequality OR Inequity AND Under-five Mortality AND Developing Countries, Gaps OR Disparity AND Unmet need for contraception AND Developing countries, Inequity OR Inequality AND Unmet need for contraception AND Developing countries, Gaps AND Disparity AND Antenatal care AND Developing countries, Inequity OR Inequality AND Antenatal care AND Developing countries, Gaps OR Disparity AND Prevention-of-Mother-To-Child-Transmission AND Developing countries, Inequality OR Inequity AND Prevention-of-Mother-To-Child-Transmission AND Developing countries, Gaps OR Disparity AND Skilled Birth Attendant AND Developing countries, Inequality OR Inequity AND Skilled Birth Attendant AND Developing countries, Gaps OR Disparity AND Stunting in children AND Developing countries, Inequality OR Inequity AND Stunting in children AND Developing countries, Gaps OR Disparity AND Postnatal Care AND Developing countries, Inequality OR Inequity AND Postnatal Care AND Developing countries, Gaps OR Disparity AND Exclusive breastfeeding AND Developing countries, Inequality OR Inequity AND Exclusive Breastfeeding AND Developing countries, Gaps OR Disparity AND DPT3 immunization AND Developing countries, Inequality OR Inequity AND DPT3 immunization AND Developing countries

Gaps OR Disparity AND Care seeking for children with suspected pneumonia AND Developing countries, Inequality OR Inequity AND Care seeking for children with suspected Pneumonia And Developing countries. The Preferred Reporting Items for Systematic Reviews and Meta- Analysis guidelines were followed in searching the results.

### Inclusion criteria

We extracted data from studies that met the following inclusion criteria: 1) Publication in peer-reviewed literature in the last 10 years (2008-2018); 2) Study must be based on data from at least one nationally representative data; 3) Study must have a clearly defined primary outcome, encompassing one or more of the eleven pre-defined core RMNCH indicator; 4) Study must have investigated the effect of at least one of three ‘dimensions of inequality’, which includes: Income level, level of education and place of residence; 5) Data from low or middle-income countries only must have been utilized; 6) Included studies must have utilized a quantitative analytical method; and 7) The statistical analysis utilized must be comparable through meta-analysis.

### Exclusion criteria

We excluded studies based on the following criteria: 1) Studies that did not use a nationally representative data; 2) Studies that were not peer-reviewed; 3) Studies that did not assess at least one of the 3 dimensions of inequality: place of residence, income level and level of education; 4) Studies that did not include at least one of the RMNCH outcome measures; 5) Studies that used data from high income countries; 6) Studies that were purely descriptive; 7) Studies that used statistical methodologies that cannot be meta-analysed

### Data extraction

**Figure 1:**
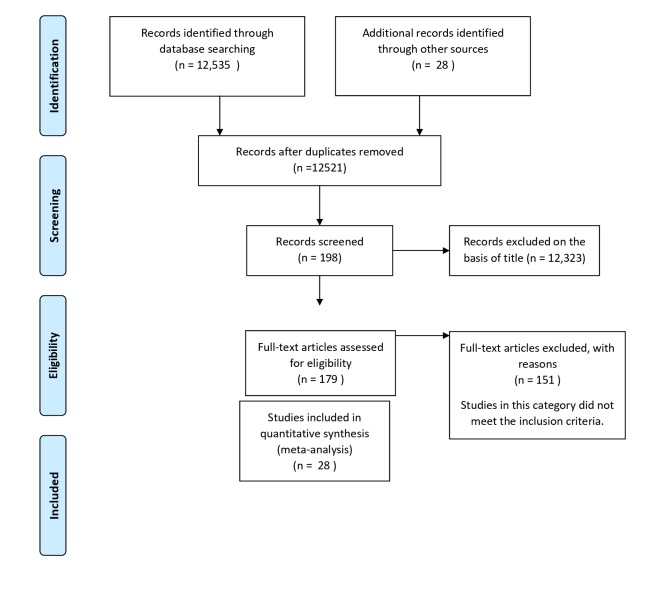
PRISMA flowchart of the included studies.

The process of identifying suitable articles is described in **Figure 1**. Two reviewers conducted initial screening of relevant articles, using a standardized form with clearly stated exclusion and inclusion criteria. The following data were collected with a standardized data collection form: 1) Data on study characteristics extracted includes: study author, publication date, country of study, source of survey utilized for the study, study design, outcome measure and dimension of inequality; 2) The study outcome comprises of nine of the eleven core indicators for which equity data was available; 3) The measure of association between the outcome (core indicators) and dimension of inequality extracted, which includes: odds ratio and the corresponding confidence interval, or standard error.

### Risk of bias assessment

We assessed publication bias using the funnel plots, risk of bias was further assessed at outcome level using the Eggers regression test, and the trim-fill analysis was used to exclude studies with significant bias with *p* value at less than 0.05. Sensitivity analysis was also conducted using one study removing at a time and cumulative removing method to determine whether the overall effect result is due to any study. The funnel plots were inspected for asymmetry.

### Quality assessment

Two independent assessors determined the quality of each included study using the Effective Public Health Practice Project’s (EPHPP) quality assessment tool. The quality was categorized into low, moderate or high, depending on the risk of bias in the study result (see Table S1 in **Online Supplementary Document**).

### Statistical analysis

Our initial search generated 12 535 abstracts and/or full articles matching our keywords. However, upon further screening, 28 studies [19-46] met the inclusion criteria. Of these 28 studies, 3 studies were of moderate scientific quality [22,26,41], the rest were of high quality [19-21,23-25,27-40,42-46]. Three studies were based on combined multi-country survey data [29,36,43], nine studies were from low middle income West African countries [19,22,23,27,28,30,38,40,45] two were from low income West African countries [33,39], five studies were from low income South-Eastern Asia countries [21,31,35,42,44, eight studies were from low income East African countries [20,25,26,32,34,37,41,46] and one study was conducted in Palestine [24] (see Table S2 in **Online Supplementary Document**).

All included studies represent a total population of 626 030 participants, we utilized the odds ratio as a comparable measure of association between the dimensions of inequality and the outcome variables. The standard error for each measure was calculated from the corresponding confidence intervals. A random effects analysis was conducted to obtain a weighted pooled odds ratio. A forest plot was then used to graphically illustrate the pooled estimates. Heterogeneity of the included studies was assessed using Q-statistic test and the I^2^ statistic (if I^2^ is less than 50%, studies will be considered homogeneous). Both Funnels plots and eggers tests for publication bias were conducted using the metafor package for R software [47]. Forest plots and pooled odd ratios for effects size were performed using Review Manager version 5.3 tool [48].

## RESULTS

### Publication bias

The result of the analysis of the effects of inequality on all outcome indicators showed minimal asymmetry. A visual inspection of the funnel plots showed no evidence of publication bias (see Figures S1-S6 in **Online Supplementary Document**). Findings from the Egger’s regression test supports the finding that there is no statistically significant evidence of publication bias for any of the included studies (**Table 1**).

**Table 1:** Eggers regression analysis

Dimension of inequality	RMNCH outcome	Eggers test *P*-value
Income	Negative indicators*	0.93
Education	Negative indicators*	0.45
Place of residence	Negative indicators*	0.45
Income	Positive indicators†	0.22
Education	Positive indicators†	0.49
Place of residence	Positive indicators†	0.40

*Negative indicators = Under five mortality fate, Stunting in under five children, Unmet need for contraception.

†Positive indicators = Antenatal care, Skilled birth attendance, Postnatal care, Exclusive breastfeeding, Three dosages of diphtheria, pertussis and tetanus immunization, Care seeking for children with suspected pneumonia.

### Effect of income level on negative RMNCH indicators

**Figure 2:**
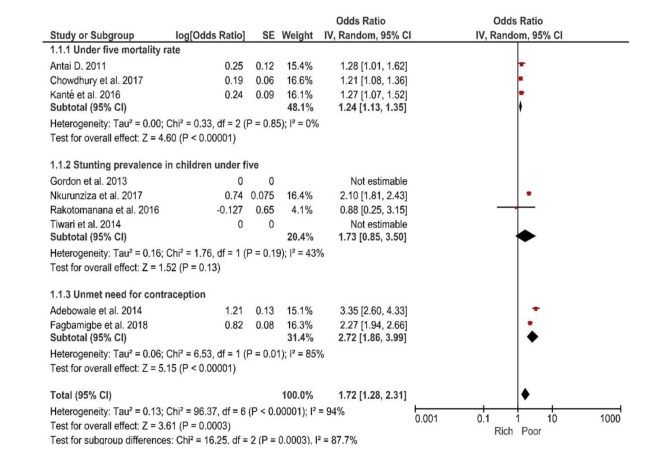
Forest plot of the effects of income level on negative indicators of Reproductive, Maternal, Newborn and Child Health (RMNCH).

A pooled estimate of comparison between the richest and poorest households was determined for U5MR, stunting in children under five years and unmet need for contraception. The pooled effect size of income on U5MR was OR = 1.24 (95% CI = 1.35-1.113), all included studies investigating U5MR were homogeneous (I^2^ = 0%, *P* < 0.00001). Similarly, regarding stunting in children under five, the poorest households were more likely to have stunted children (OR = 1.73, 95% CI = 0.85-3.5), (I^2^ = 43%, *P* = 0.13). Finally, women living in poor households were twice likely to have unmet need for contraception (OR = 2.72, 95% CI = 1.86-3.99), (I^2 =^  85%, *P* = 0.00001) compare to those in richer households. Studies included in the assessment of effect of income on stunting prevalence were homogeneous, while those assessing the effect of income on unmet need for contraception were heterogenous (**Figure 2**).

### Effect of level of education on negative indicators of RMNCH

**Figure 3:**
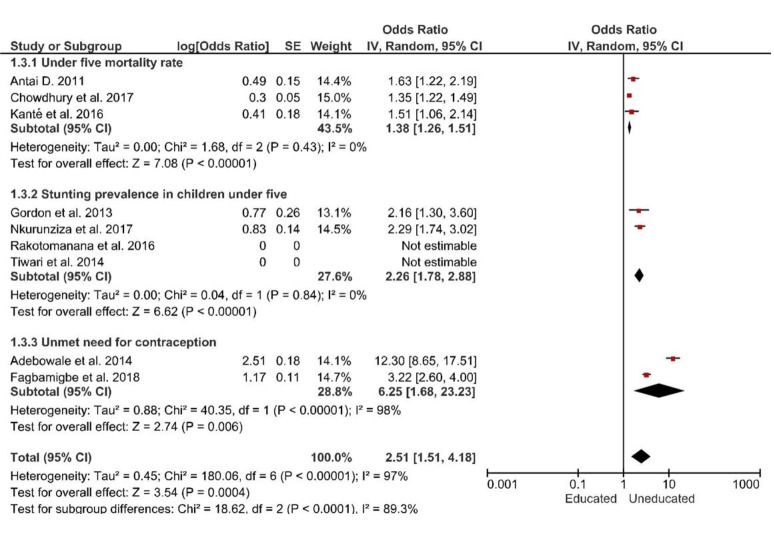
Forest plot of the effects of level of education on negative indicators of Reproductive, Maternal, Newborn and Child Health (RMNCH).

Regarding the level of education, comparisons were made between those with no formal education, or primary education and those with secondary or higher level of education. We found marked variation in U5MR, stunting in children and unmet need for contraception based on level of education. The pooled odd ratio for U5MR was OR = 1.38 (95% CI = 1.26-1.51; I^2 =^  0%, *P* < 0.0001) while the pooled odd ratio for stunting in children under five years and unmet need for contraception were: OR = 2.26 (95% CI = 1.78-2.88; I^2 =^  0%, *P* < 0.0001) and OR = 6.25 (95% CI = 1.68-23.23; I^2 =^  98%, *P* = 0.006) respectively. Studies assessing U5MR and stunting were homogeneous, while those assessing unmet need for contraception were heterogenous (**Figure 3**).

### Effect of place of residence on negative indicators of RMNCH

**Figure 4:**
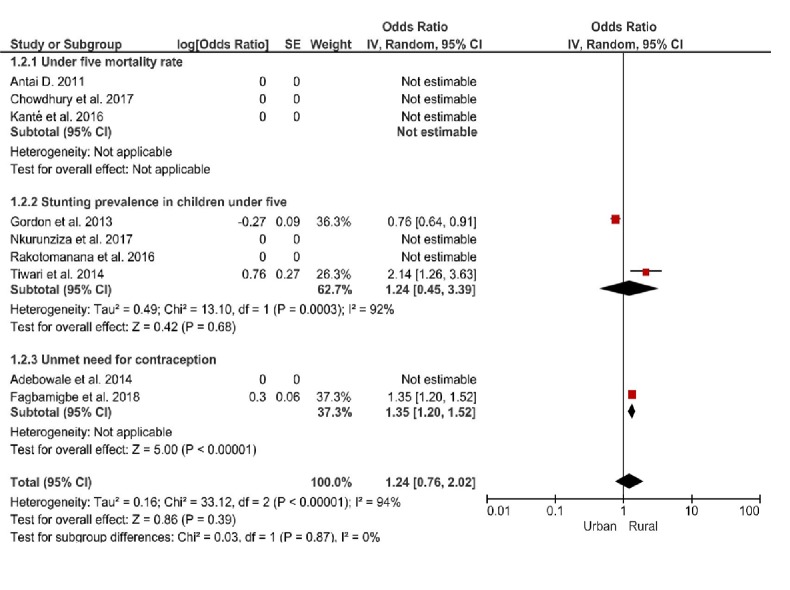
Forest plot of the effects of place of residence on negative indicators of Reproductive, Maternal, Newborn and Child Health (RMNCH).

Regarding place of residence, very minimal rural-urban gaps were identified with regards to stunting in children under five years and unmet need for contraception. A pooled estimate could not be derived for effect of place of residence on U5MR, as rural-urban differences were not assessed in any of our included studies. We found that children under five residing in rural areas were relatively more likely to be stunted (OR = 1.24, 95% CI = 0.45 -3.39; I^2^ = 92%, *P* = 0.68) and women were more likely to have unmet need for contraception (OR = 1.35, 95% CI = 1.20-1.52; *P* ≤ 0.00001), than their urban counterparts (**Figure 4**).

### Effect of income level on positive indicators of RMNCH

**Figure 5:**
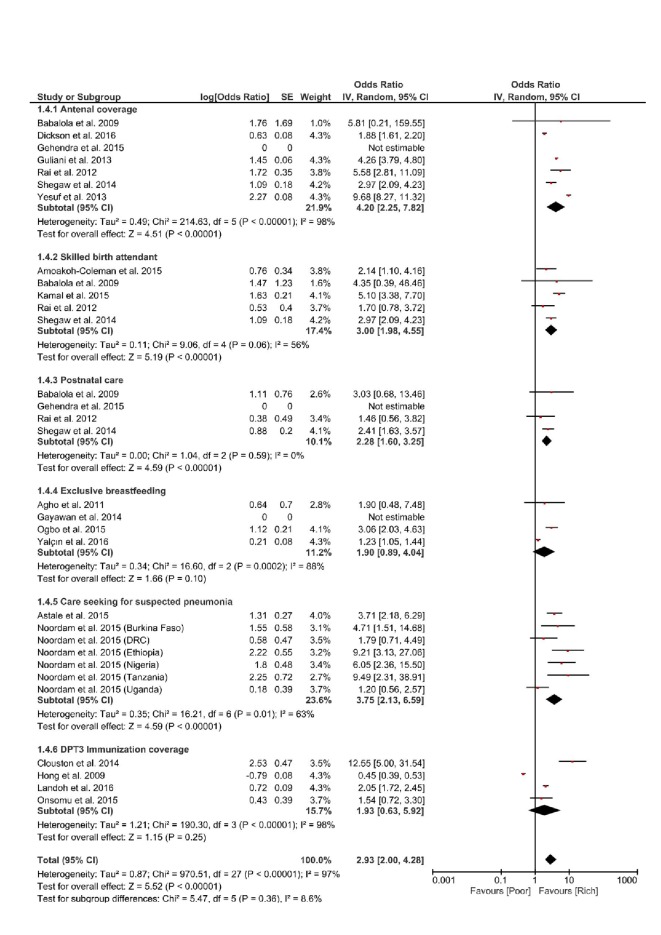
Forest plot of the effects of income level on positive indicators of Reproductive, Maternal, Newborn and Child Health (RMNCH).

Regarding the positive indicators of RMNCH, a pooled estimate was derived for the effect of income level on ANC, SBA, PNC, exclusive breastfeeding, DPT3 immunization and care seeking for suspected pneumonia. Comparisons were made between the poorest and richest households. The richest household had better assess or coverage on all these indicators, the most significant effect of income disparity was on ANC and care seeking for suspected pneumonia in children, pooled estimates were OR = 4.20 (95% CI = 2.25–7.82; I^2^ = 98%, *P* < 0.00001) and OR = 3.75 (95% CI = 2.13-6.59; I^2 =^  63%, *P* = 0.01) respectively. The heterogeneity was significant for both indicators. (**Figure 5**).

### Effect of level of education on positive indicators of RMNCH

There were disparities based on the level of education, when comparing households with no formal, or primary level of education, with those with secondary or higher level of education.

**Figure 6:**
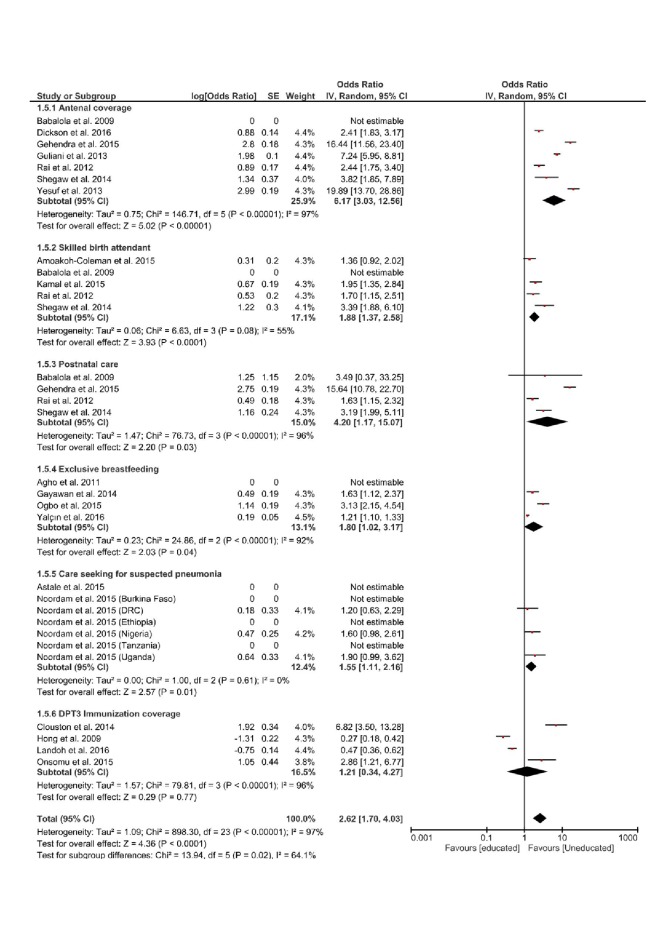
Forest plot of the effects of level of education on positive indicators of Reproductive, Maternal, Newborn and Child Health (RMNCH).

Meta-analysis showed that of all positive indicators assessed, education had the most significant effect on ANC. The most educated were 6 times more likely to have better coverage, than those that were uneducated or with primary level of education (OR = 6.17, 95% CI = 3.03-12.56; I^2^ = 97%, *P* < 0.00001). The included studies were heterogeneous (**Figure 6**).

### Effect of place of residence on positive indicators of RMNCH

**Figure 7:**
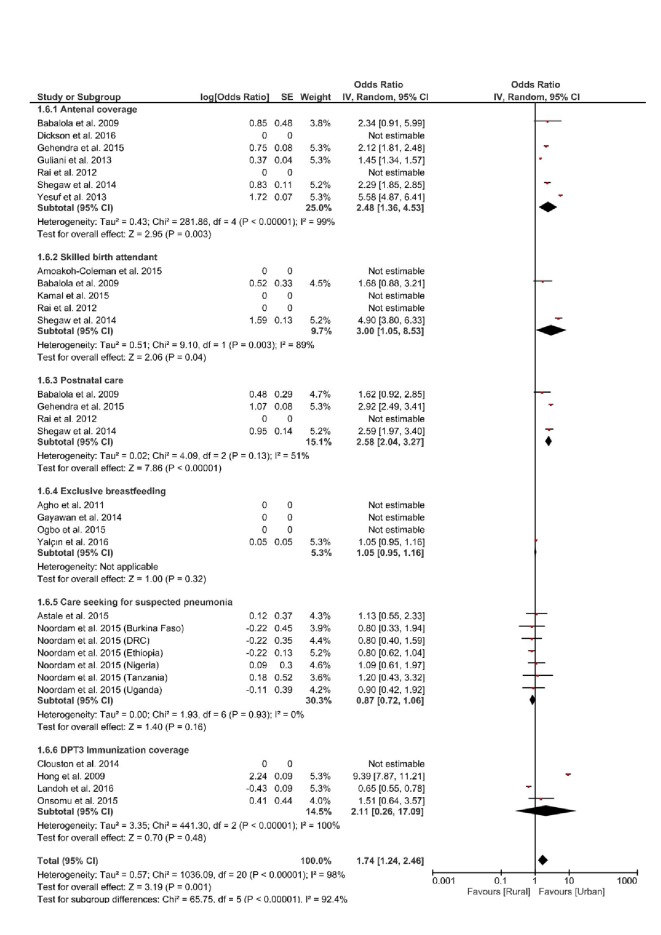
Forest plot of effect of place of residence on positive indicators of Reproductive, Maternal, Newborn and Child Health (RMNCH).

Inequality based on place of residence was assessed by comparing RMNCH coverage for those in urban areas, with those in rural areas. Meta-analysis of the effect of place of residence on positive indicators of RMNCH, showed that the most significant urban-rural gap was with respect to the use of skilled birth services, which favoured those in the urban area, they were 3 times more like to use skilled services for delivery compared to those in rural areas (OR = 3.00, 95% CI = 1.05 -8.53; (I^2 =^  98%, *P* = 0.04). The included studies were heterogenous (**Figure 7**).

In summary, women living in poor households were twice likely to have unmet need for contraception compared to those in richer households, similarly uneducated women were six times more likely to have unmet need for contraception than those that have secondary or higher level of education, children from households with the lowest level of education were twice as likely to be stunted than those from more educated households. In contrast, women in the richest households were 4 times more likely to receive antenatal care, and to seek care for children with suspected pneumonia, equally women with secondary or higher level of education were 6 times more likely to receive antenatal care. Finally, women in urban area were 3 times more likely to use skilled services for delivery, when compared with those in rural areas.

## DISCUSSION

Overall, we have examined existing evidence regarding the effects of income, education and place of residence on RMNCH indicators, with focus on nationally representative studies. In general, we found strong and consistent evidence that the negative indicators were pro-poor, while the positive indicators were considerably pro-rich. This implies that poorer households were likely to have unmet need for contraception, higher U5MR, stunted under-five children, while those in richer, educated households, were more likely to receive ANC, have skilled delivery, receive PNC, exclusively breastfeed their children.

The children in the richer households were also likely to complete three dosages of the DPT vaccine. These same trends also largely applied to those in the urban areas, apart from a rather striking exception in care seeking for suspected pneumonia, we found that caregivers in urban households were less likely to seek care for suspected pneumonia, when compared to residents in rural areas. This finding on care seeking for suspected pneumonia however will benefit from further investigation, as other evidence from literature suggests that children in rural area are less likely to be treated for suspected pneumonia [49]. Moreover, caregivers in rural areas are also likely to be poor and uneducated [49,50]. The incongruence in our finding, may however be due to the unreliability of verbal survey responses given by caregivers regarding their care seeking behaviour, for children with pneumonia [51]. A study by Zhang et al., also shows that, while caregivers in rural communities may indeed actively seek for care, very few children were getting the appropriate treatment [52].

We found that disparity in income levels had the most significant effect on ANC, such that the richest subgroup were 4 times likely to use ANC services when compared to the poorest; (see **Figure 5**). This finding is in line with those in the recent World Health Organization (WHO) state of inequality report, which found a 10-percentage point difference between the richest and the poorest, for ANC coverage (at least one visit) in 84 LMICs countries [6]. Similarly, income levels had significant effect on care seeking for children with suspected pneumonia infection, the richest were about 4 times likely to seek care when compared to the poorest sub-group. This finding appears congruent with other findings in the literature, suggesting that the greatest level of disparities is usually seen with regards to interventions requiring fixed health facilities, as opposed to community level interventions such as immunization [53].

Overall, level of education had the most significant effect on both unmet need for contraception, and antenatal coverage. For both respective indicators, the most educated were 6 times more likely to have better coverage, than those that are uneducated or with primary level of education. Educated women in LMICs have been found to be relatively more empowered and have more autonomy, than their uneducated counterparts [3]. In addition, they have a better understanding of the need to utilise health care services to mitigate against the risk of health complications [6]. These may help explain our findings regarding the effect of education.

In this study, we found that urban-rural inequality had the most significant effect on the use of skilled birth services. Those in the urban area, were 3 times more like to use skilled services for delivery compared to those in rural areas. Provision of health care services in rural areas are often impaired by geographical barriers, more so women living in rural areas may have less socio-economic opportunities and may be poorer than those in urban areas [49].

### Implication and recommendation for future studies

Achieving equity in RMNCH coverage remains a pressing challenge, however as identified in other studies and reports, significant progress has been made over the past few decades [7]. This progress may have accounted for some of the very limited or narrow disparities seen with respect to some indicators in our study. For example, the least disparity was with regards to DPT3 immunization (see **Figure 6**). These narrowing disparities may allude to some level of progress achieved from many decades of concerted efforts to promote uptake of immunization especially in LMICs, although coverage levels have also stagnated in recent years [54].

Our study further shows the need to focus on promoting equity in aspects of continued reproductive care such as ANC, Skilled birth services and PNC. The inclusion of these reproductive services within a Universal health care (UHC) plan needs to be prioritized in LMICs.

Finally, disaggregating maternal mortality estimates for equity breakdown are of vital importance, similarly there is a need for data with an equity breakdown of ARV prophylaxis coverage among pregnant women with HIV. The design of our study did not allow us to measure changes or progress made over time, hence there is a need for more studies measuring both absolute and relative change in inequality over time, all of which must be underpinned by household level disaggregation, in line with the goals and targets of the sustainable development goals (SDG).

### Limitation of the study

We only analysed a selection of RMNCH indicators, there are several other RMNCH indicators; all of which could not be included in this study. The scope of our study was also limited to studies measuring association between selected indicators and equity stratifiers through regression coefficient, several RMNCH equity studies often measure coverage using other analytical approaches such as ratios, differences, slope index of inequality, and concentration index and population attributable risk. Finally, we were unable to analyse certain indicators, due to lack of consistent and comparable data.

## CONCLUSIONS

In conclusion, this study has provided a pooled evidence on equity and RMNCH, we have assessed the effects of inequality across nine RMNCH interventions and outcomes, most importantly we found significant disparity regarding unmet need for contraception, use of skilled birth services, antenatal care, and care seeking for children with suspected pneumonia. The least level of disparity was found in regard to number of children completing 3 doses of DPT vaccine, this reflects the progress that has been made in the area of children immunization at community and household levels. In contrast, there is still a lot to be done in providing affordable and accessible services along the continuum of reproductive care; antenatal care, skilled birth services, and postnatal care. Our study also indicates that a lot still needs to be done in reducing the levels of unmet need for contraception in LMICs. We hope that our findings will provide evidence-based information to policy makers in LMICs on aspects of RMNCH services that needs to be prioritised.

## Additional material

Online Supplementary Document
