# Supplementary material for: Systematic review and meta-analysis of the association between dimensions of inequality and a selection of indicators of Reproductive, Maternal, Newborn and Child Health (RMNCH)
Source: J Glob Health. 2019 May 12;9(1):010429. doi: 10.7189/jogh.09.010429 (PMC6513502; doi:10.7189/jogh.09.010429)

J Glob Health 2019; 9: 010429

Table S1: Study quality assessment using EHPP assessment tool.

Study	Selection bias	Study design	Confounder	Blinding	Data collection	Withdrawals & Drop outs	Study Quality
Antai D.[34] (2011)	Strong	Cross sectiona	Strong	Moderate	Strong	Strong	Strong
Chowdhury et al.[38] (2017)	Strong	Cross sectional	Moderate	Moderate	Strong	Strong	Strong
Kanté et al.[35] (2016)	Weak	Longitudinal study	Moderate	Strong	Strong	Strong	Moderate
Yesuf et al.[14] (2013)	Strong	Cross sectional study	Moderate	Strong	Strong	Strong	Strong
Guliani et al.[37] (2013)	Strong	Cross sectional study	Strong	Strong	Strong	Strongh	Strong
Dickson et al.[13] (2016)	Strong	Cross sectional	Strong	Strong	Strong	Strong	Strong
Babaloloa et al.[39] (2009)	Strong	Cross sectional	Strong	Strong	Strong	Strong	Strong
Shegaw et al.[40] (2014)	Strong	Cross sectional	Strong	Strong	Strong	Strong	Strong
Gehendra et al. [15](2015)	Strong	Cross sectional	Moderate	Strong	Strong	Strong	Strong
Rai et al.[16] (2012)	Weak	Cross sectional	Moderate	Strong	Strong	Strong	Moderate

Study	Selection bias	Study design	Confounder	Blinding	Data collection	Withdrawals & Drop outs	Study Quality
Gordon et al.[18] (2013)	Strong	Cross sectional	Moderate	Strong	Strong	Strong	Strong
Tiwari et al.[36] (2014)	Strong	Cross sectional	Strong	Strong	Strong	Strong	Strong
Nkurunziza et al.[19] (2017)	Strong	Cluster-randomized controlled trial	Strong	Strong	Strong	Strong	Strong
Rakotomanana et al.[20] (2016)	Strong	Cross sectional	Weak	Strong	Strong	Strong	Moderate
Ogbo et al.[21] (2015)	Strong	Cross sectional	Strong	Strong	Strong	Strong	Strong
Gayawan et al.[22] (2014)	Strong	Cross sectional	Moderate	Strong	Strong	Strong	Strong
Yalcin et al.[23] (2016)	Strong	Cross sectional	Strong	Strong	Strong	Strong	Strong
Agho et al.[24] (2011)	Strong	Cross sectional	Strong	Strong	Strong	Strong	Strong
Onsomu et al.[26] (2015)	Strong	Cross sectional	Strong	Strong	Strong	Strong	Strong
Landoh et al.[27] (2016)	Strong	Cross sectional	Strong	Strong	Strong	Strong	Strong
Clouston et al.[28] (2014)	Strong	Cross sectional	Moderate	Strong	Strong	Strong	Strong
Hong et al.[29] (2009)	Strong	Cross sectional	Strong	Strong	Strong	Strong	Strong
Noordam et al.[30] (2015)	Strong	Cross sectional	Strong	Strong	Strong	Strong	Strong

Study	Selection bias	Study design	Confounder	Blinding	Data collection	Withdrawals & Drop outs	Study Quality
Astale et al.[31] (2015)	Strong	Cross sectional	Strong	Strong	Strong	Strong	Strong
Adebowale et al.[33] (2014)	Strong	Cross sectional	Moderate	Strong	Strong	Strong	Strong
Fagbamigbe et al.[32] (2014)	Strong	Cross sectional	Strong	Strong	Strong	Strong	Strong
Amoakoh-coleman et al.[17] (2015)	Strong	Cross sectional	Strong	Strong	Strong	Strong	Strong
Kamal et al.[25] (2015)	Strong	Cross sectional	Moderate	Strong	Strong	Strong	Strong

Table S2. Characteristics of the included studies

Author	Country	Source of data	Outcome	Dimension of Inequality
Antai D.[34] (2011)	Nigeria	DHS	Under five mortality	Wealth index, Education.
Chowdhury et al.[38] (2017)	Bangladesh	DHS	Under five mortality	Asset score, Education level,
Kanté et al.[35] (2016)	Tanzania	DHS	Under five mortality	Wealth quintiles, Education.
Yesuf et al.[14] (2013)	Ethiopia	DHS	ANC	Wealth index, Education
Guliani et al.[37] (2013)	32 countries	DHS	ANC	Education, Wealth index, Residence.
Dickson et al.[13] (2016)	Ghana	DHS	ANC	Residence, Education, Wealth index.
Babaloloa et al.[39] (2009)	Nigeria	NARHS[a]	ANC, PNC, SBA	Education, SES, Residence
.				
Shegaw et al.[40] (2014)	Ethiopia	DHS	ANC, PNC, SBA	Residence, Education, Wealth index.

Gehendra et al.[15] (2015)	Nepal	DHS	ANC, PNC, SBA	Age, Residence, Education.

a = National HIV/AIDS and reproductive health survey

Table S2 contd. Characteristics of the included studies

Author	Country	Source of data	Outcome	Dimension of Inequality
Rai et al.[16] (2012)	Nigeria	DHS	ANC, PNC, SBA	Study focused on Adolescents – Residence, Education, wealth quintile.
Gordon et al.[18] (2013)	Palestine	PAPFAM[b] & MICS	Stunting in under five	Mother's Education, Place of residence.
Tiwari et al.[36] (2014)	Nepal	DHS	Stunting in under five	Education, Wealth index, Residence.
Nkurunziza et al.[19] (2017)	Burundi	PBF[c] scheme	Stunting in under five	Socio-economic status, Education.
Rakotomanana et al.[20] (2016)	Madagascar	DHS	Stunting in under five	Wealth index, Residence.
Ogbo et al.[21] (2015)	Nigeria	DHS	Exclusive breastfeeding	Education, Household wealth.
Gayawan et al.[22] (2014)	Nigeria	DHS	Exclusive breastfeeding	Education.
Yalcin et al.[23] (2016)	27 Sub-Saharan African countries	DHS	Exclusive breastfeeding	Education, Wealth index, Residence.
Agho et al.[24] (2011)	Nigeria	DHS	Exclusive breastfeeding	Education, Wealth index, Residence.

Onsomu et al.[26] (2015)	Kenya	DHS	DPT immunization	Residence, Education, Wealth index.

b = Pan Arab Project for Family Survey, c = Performance based financing scheme (survey was derived from the impact evaluation of the PBF scheme)

Table S2 contd. Characteristics of the included studies

Author	Country	Source of data	Outcome	Dimension of Inequality
Landoh et al.[27] (2016)	Togo	MICS	DPT immunization	Residence, Education
.				
Clouston et al.[28] (2014)	Madagascar	DHS	DPT immunization	Education, Wealth index.
Hong et al.[29] (2009)	Cambodia	DHS	DPT immunization	Wealth quintile, Residence, Mother's education.
Noordam et al.[30] (2015)	6 SSA countries	DHS & MICS	Care seeking for pneumonia	Residence, Parent's education, Wealth index.
Astale et al.[31] (2015)	Ethiopia	DHS	Care seeking for pneumonia	Residence, wealth index, Education.
Adebowale et al.[33] (2014)	Burkina Faso	DHS	Unmet need for contraception	Wealth quintile, education.
Fagbamigbe et al.[32] (2014)	Nigeria	DHS	Unmet need for contraception	Residence, education, Wealth quintile.
Amoakah-coleman et al.[17] (2015)		DHS	SBA	Wealth status, education, residence
Kamal et al.[25] (2015)		DHS	SBA	Education, Wealth index.

Figure S1. Funnel plot of studies assessing effect of income level on negative indicators of RMNCH.

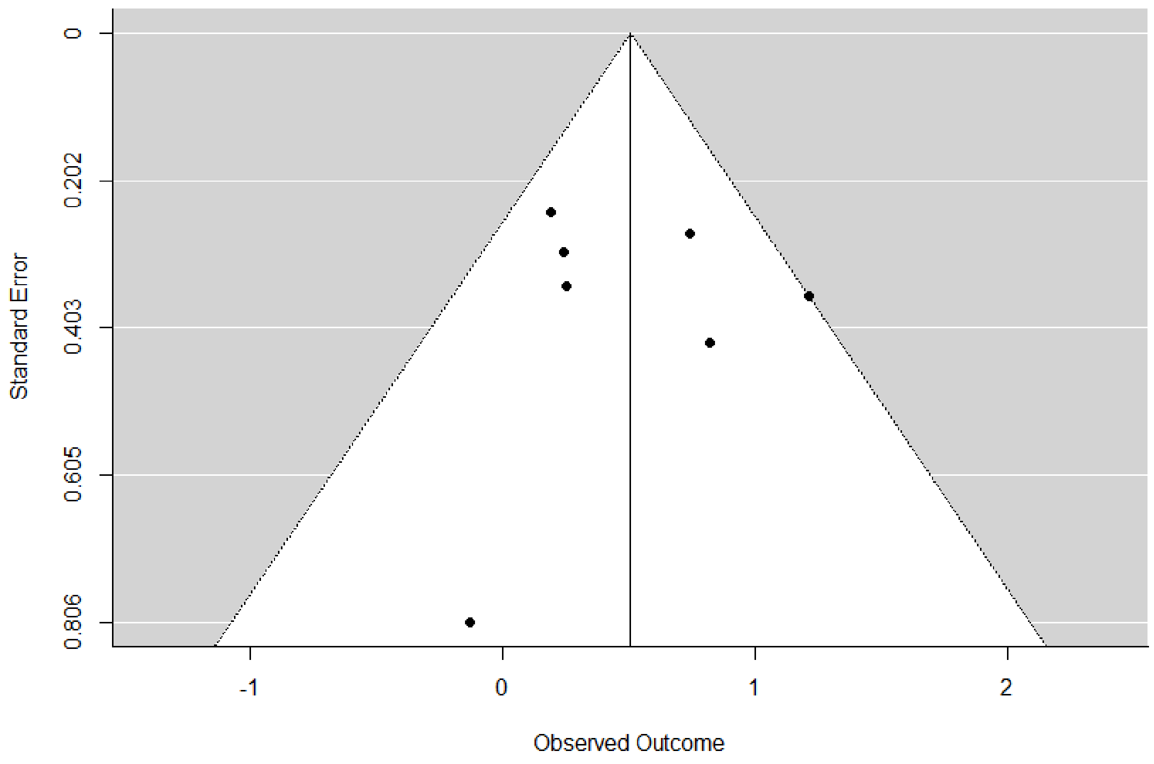

Figure S2: Funnel plot of studies assessing effect of level of education on negative indicators of RMNCH.

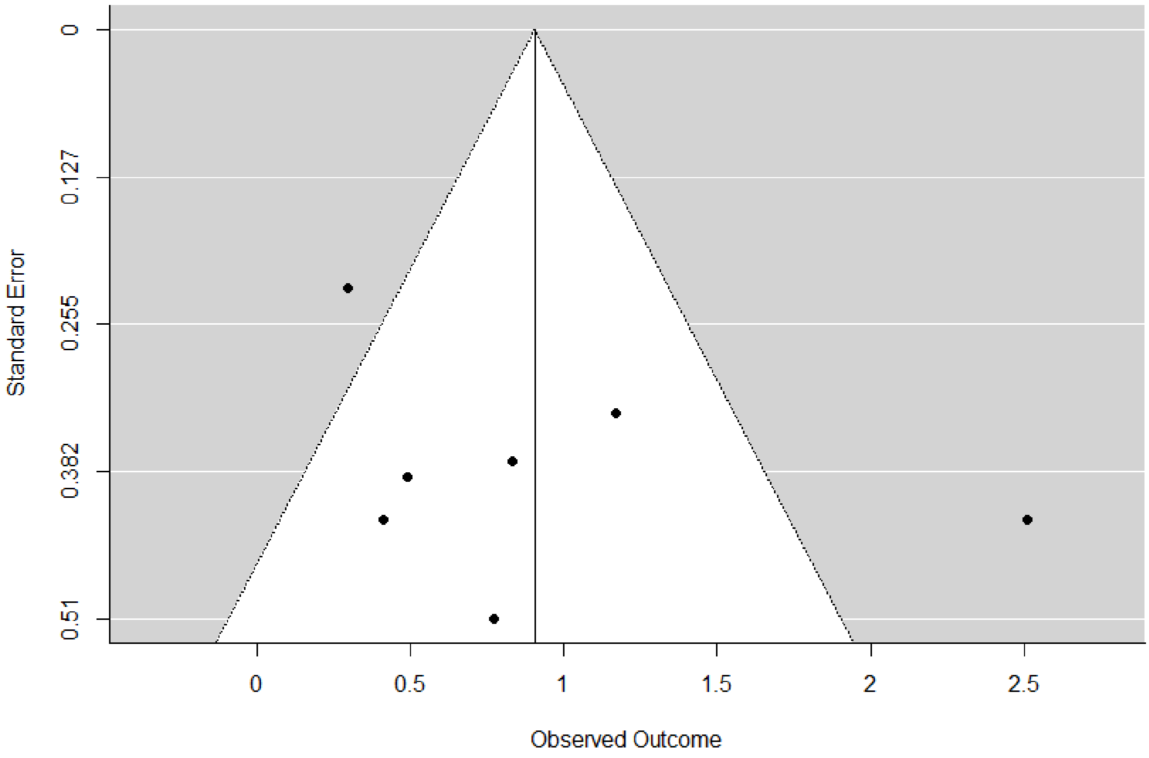

Figure S3: Funnel plot of studies assessing effect of place of residence on negative indicators of RMNCH.

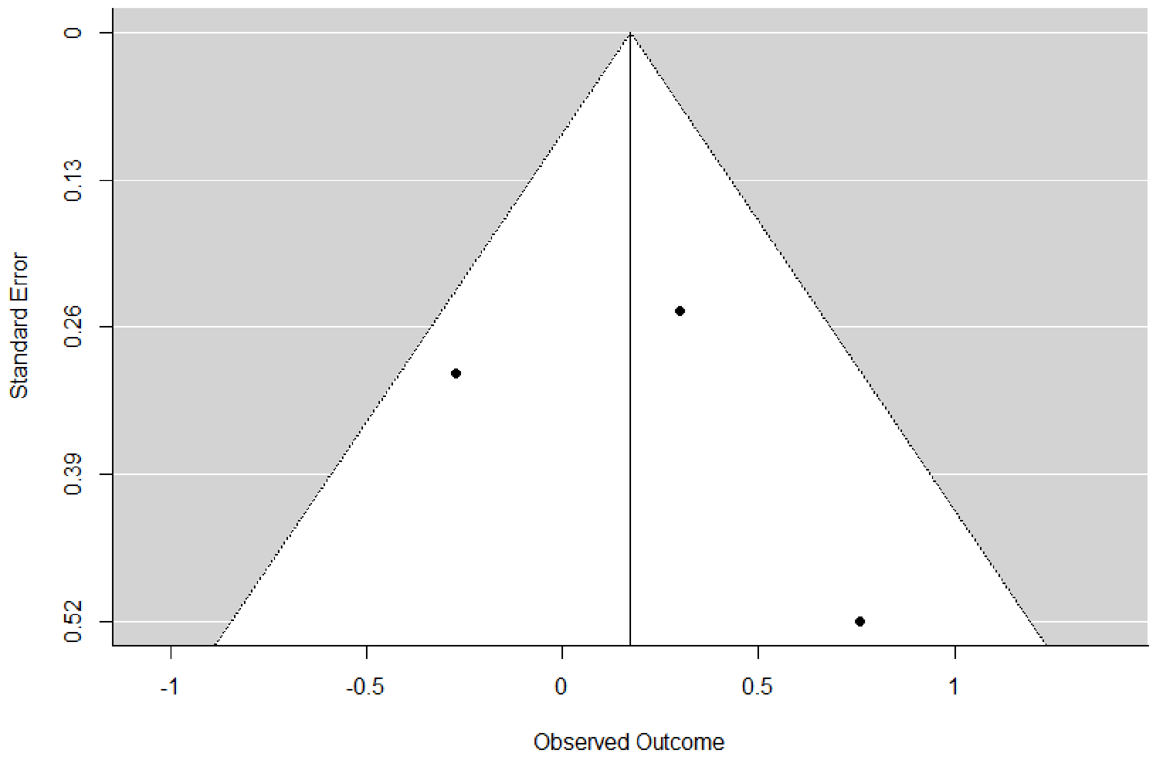

Figure S4: Funnel plot of studies assessing the effect of income level on positive indicators of RMNCH.

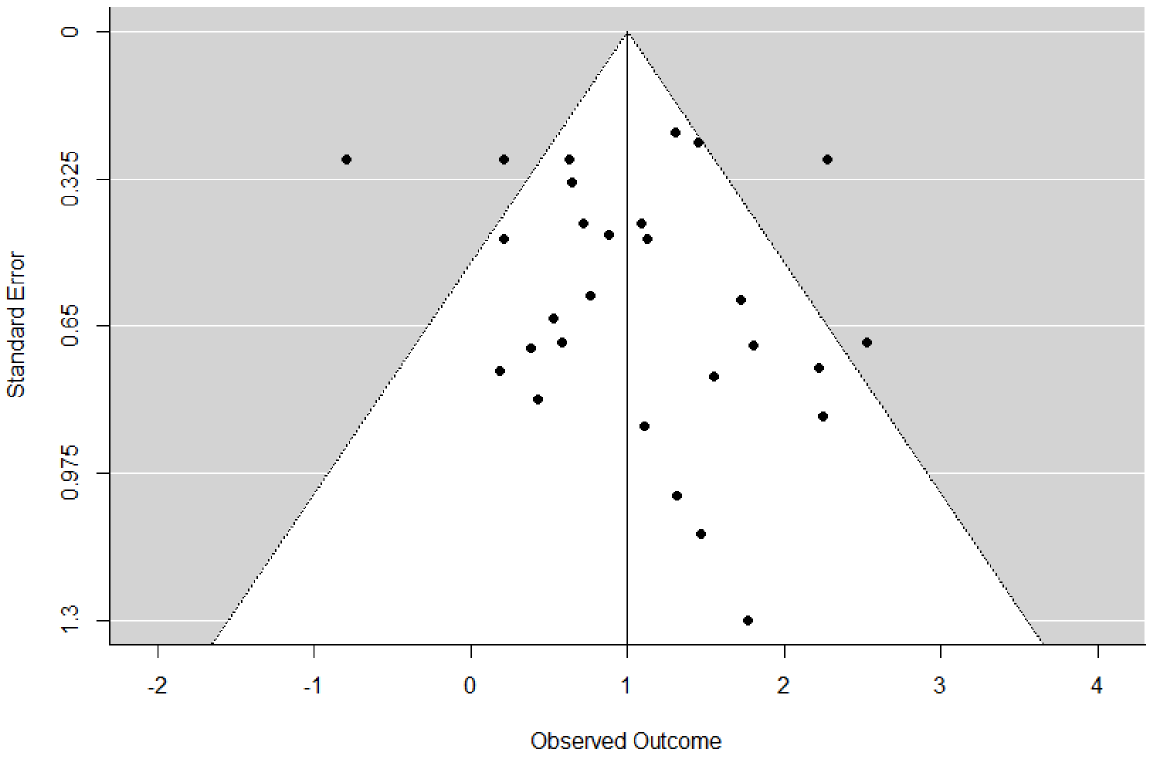

Figure S5: Funnel plot of studies assessing the effect of level of education on positive indicators of RMNCH.

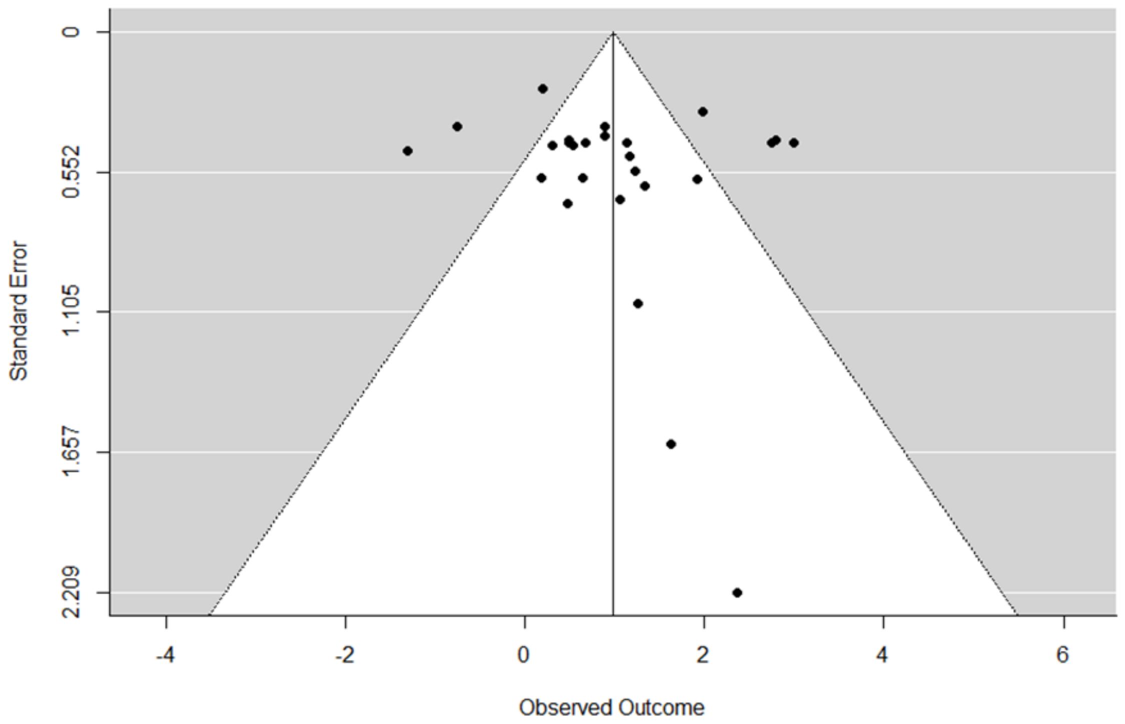

Figure S6: Funnel plot of studies assessing the effect of place of residence on positive indicators of RMNCH.

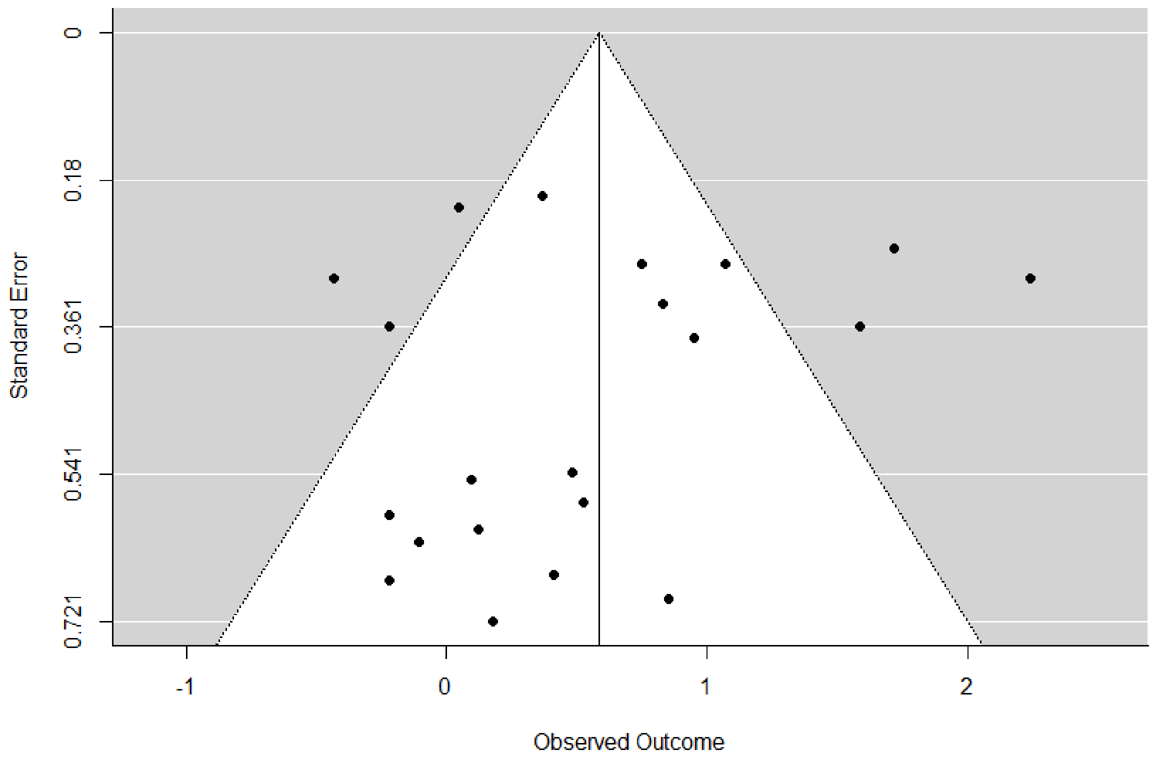

Supplement: Online Supplementary Document [file jogh-09-010429-s001.pdf]
